# Use of short message service (SMS) to improve malaria chemoprophylaxis compliance after returning from a malaria endemic area

**DOI:** 10.1186/1475-2875-8-236

**Published:** 2009-10-23

**Authors:** Lénaïck Ollivier, Olivier Romand, Catherine Marimoutou, Rémy Michel, Corinne Pognant, Alain Todesco, René Migliani, Dominique Baudon, Jean-Paul Boutin

**Affiliations:** 1 Institut de médecine tropicale du Service de santé des armées, Département d'épidémiologie et de santé publique Sud, Marseille, France; 2 Cabinet médical du 27^e ^Bataillon de chasseurs alpins, Cran Gevrier, France; 3 Département d'épidémiologie et de santé publique Nord, Ecole du Val de Grâce Saint-Mandé, France; 4 Direction régionale du Service de santé des armées, Lyon, France

## Abstract

**Background:**

Malaria chemoprophylaxis compliance is suboptimal among French soldiers despite the availability of free malaria chemoprophylaxis and repeated health education before, during and after deployment to malaria endemic areas.

**Methods:**

In 2007, a randomized controlled study was performed among a cohort of French soldiers returning from Côte d'Ivoire to assess the feasibility and acceptability of sending a daily short message service (SMS) reminder message via mobile device to remind soldiers to take their malaria chemoprophylaxis, and to assess the impact of the daily reminder SMS on chemoprophylaxis compliance. Malaria chemoprophylaxis consisted of a daily dose of 100 mg doxycycline monohydrate, which began upon arrival in Côte d'Ivoire and was to be continued for 28 days following return to France. Feasibility and acceptability were assessed by questionnaire. Cohort members were followed for a 28 day period, with compliance assessed by use of an electronic medication monitoring device, from which several indicators were developed: daily proportion of compliant individuals, average number of pills taken, and early discontinuation.

**Results:**

Among 424 volunteers randomized to the study, 47.6% were assigned to the SMS group and 52.3% to the control group. Approximately 90% of subjects assigned to the SMS group received a daily SMS at midday during the study. Persons of the SMS group agreed more frequently that SMS reminders were very useful and that the device was not annoying. Compliance did not vary significantly between groups across the compliance indicators.

**Conclusion:**

SMS did not increase malaria chemoprophylaxis compliance above baseline, likely because the persons did not benefit from holidays after the return and stayed together. So the reminder by SMS was noted by all subjects of the study. Another study should be done to confirm these results on soldiers going on holidays from employment after return or with individual travellers.

## Background

After several years of rising incidence, imported malaria is now decreasing in Europe and North America despite an increase in travel to tropical areas [1]. Yet, imported malaria remains a concern in France. The compliance of travellers with malaria chemoprophylaxis is often suboptimal. French soldiers are no exception, despite the availability of free malaria chemoprophylaxis and repeated health education sessions before, during and after deployment to malaria endemic areas [2-5]. In 2006, 54.2% of malaria cases notified to the French Defence Medical Service occurred among soldiers who had not regularly taken their chemoprophylaxis within the eight days prior to its onset [6].

An unpublished study performed in 2004 showed that 45.7% of French soldiers returning from malaria endemic areas were noncompliant with their daily chemoprophylaxis on at least two consecutive days during the 28 days following their return. Bachelor soldiers were at higher risk of poor compliance for malaria chemoprophylaxis, while soldiers with relatives or friends to regularly remind them to take their chemoprophylaxis were at lower risk of noncompliance. Based on these observations, an experimental study was set up in 2007 to employ daily SMS (short message system) messages to remind French soldiers returning from a malaria endemic area to take their chemoprophylaxis. The aims of the study were to assess the feasibility and acceptability of sending a daily SMS reminder message via mobile device to remind soldiers to take their malaria chemoprophylaxis, and to assess the impact of the daily reminder SMS on chemoprophylaxis compliance. In this paper, the authors report the results of this study.

## Methods

### Study population

A battalion of 492 French soldiers was engaged in peace-keeping operations during a four-month period (from March to July 2007). Officers, non-commissioned officers and privates of this company were deployed to Man, a small town located in the centre of Côte d'Ivoire, where they resided under low-level sanitary conditions.

The malaria chemoprophylaxis regimen consisted of a daily dose of 100 mg doxycycline monohydrate, starting on arrival in Côte d'Ivoire, and to be continued for 28 days following return to France. This prospective study was performed during the 28-day period (D+1 through D+28) following return, from July to August 2007.

Among the group returning from Côte d'Ivoire during this period, study inclusion criteria were defined as: volunteering for the study; possessing a mobile device capable of receiving SMS; and remaining within reach of SMS messaging capability during the prospective 28-day period.

### Power calculations

Based on the previous study showing 45.7% discontinuation in daily prophylaxis during the 28 days following return, the control group receiving only standard health information on return was expected to be 45% compliant. In the SMS group, a 33% increase in compliance was expected (i.e. 60% compliance). With α risk equal to 5% and a 1-β power equal to 80%, the number of subjects required to be assigned to each of the SMS and control groups was determined to be 186 by EPI-INFO 3.3.2^® ^software.

### Randomization

Several air flights were necessary to return all soldiers of the battalion to France. From each flight, a listing of returning soldiers, along with their rank, name and forename, age, gender and mobile phone number, was provided to the French medical service. Soldiers without cell phone were excluded, and remaining study inclusion criteria were assessed upon arrival, with randomization subsequently performed by assigning to each soldier, a random number from a uniform distribution between 0 and 1 generated using Excel^® ^software. If the number was superior or equal to 0.5, the soldier was assigned to the SMS group; otherwise, the soldier was assigned to the control group.

### SMS group message

The mobile device numbers of subjects enrolled to the SMS group were provided to a commercial SMS messaging service, SMS Box^®^, to facilitate the sending of a standardized daily message, in French, during the 28 days following return, at midday hour: "Remember to take your doxycycline pill at midday. In case of fever, consult a physician and tell him you have recently returned from Côte d'Ivoire". Members of the control group received no SMS messages as part of this study during the same period.

### Assessing compliance

An electronic medical monitoring device, referred to as MEMs^® ^(Medication Event Monitoring system, Aardex Ltd, Vise, Belgium) was issued to each study subject and used to assess compliance. MEMs^® ^consist of time and date-stamping microcircuitry incorporated into drug packaging, providing a continuous record of timing of presumptive doses. Its interpretation as a dosing record is based on three assumptions: (i) correct dosing is taken at each opening, (ii) correct dosing occurs immediately after the opening (iii) the monitored package is the patient's only source of drug.

### Acceptability and feasibility

Questions pertaining to acceptability and feasibility, as well as other factors known to be associated to compliance (previous stay in malaria endemic areas, reminders by other persons to take malaria chemoprophylaxis) were assessed via a self-administered questionnaire completed at the end of the 28-day study.

### Compliance indicators

A subject was considered compliant with chemoprophylaxis on a given day if the MEMs^® ^indicated they had taken a doxycycline pill on that day. If not, they were considered non-compliant on that day. Several indicators were defined to assess the level of compliance. The primary indicator was the proportion of fully compliant subjects (e.g. the proportion of subjects who took a pill every day during the entire duration of the study). Several secondary indicators were also defined, including the average number of pills taken and the daily proportion of compliant persons. Additionally, as the half-life of doxycycline is about 24 hours and protection against malaria wanes after two missed doses, an early discontinuation of malaria chemoprophylaxis was defined as the start of at least two consecutive days missed chemoprophylaxis, with or without subsequent resumption.

### Duration of study

The MEMs^® ^were distributed to study participants following arrival in France from Côte d'Ivoire. Depending on the hour of return from Côte d'Ivoire, some received MEMs^® ^the day of their return (D+1) and some the day after (D+2). To obtain comparable data, malaria chemoprophylaxis compliance was analysed during 27 days between D+2 and D+28. For analysis of survival data, subjects presenting with malaria after returning were censored at the day of onset of malaria.

### Comparisons with previous data

An observational study using similar questionnaire and MEMs^® ^data to assess malaria chemoprophylaxis compliance was performed among company of 116 French soldiers returning from Côte d'Ivoire in 2004 (unpublished data). In this earlier study, subjects received only health education sessions base. Comparisons have been made between data collected in this study and those collected in the group without SMS in the present study.

### Statistical analysis

MEMs^® ^were read using Power view^® ^software (Aardex Ltd, Vise, Belgium). Statistical analysis was performed using SAS 9.1^® ^software (SAS Institute, NC, Carry, USA). To take into account the non respect of the risk proportionality hypothesis during time, a survival analysis with a Weibull model was performed [7]. Used in the analysis of survival data, the Weibull model provides an alternative, fully parametric approach to the Cox model. In addition to being proportional, it is simultaneously an accelerated failure-time model and is the only parametric distribution to possess both properties.

## Results

Among the 492 soldiers returning from Côte d'Ivoire, 424 (86.2%) met inclusion criteria and were randomized to either the control group or the SMS group. The randomization method resulted in a misbalance between groups: 202 (47.6%) were assigned to the SMS group, and 222 (52.3%) to the control group.

**Figure 1:**
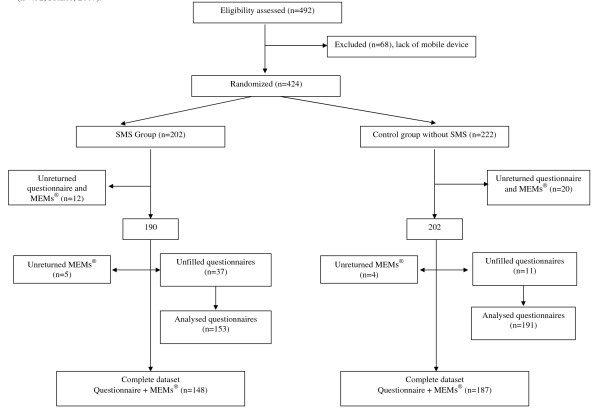
**Acceptability, feasibility, and impact on malaria chemoprophylaxis compliance of reminder SMS: overview of study design and participation (n = 492, France, 2007)**.

81.1% of subjects (344/424) returned questionnaires at the conclusion of the study: 44.5% (153/344) among the SMS group, and 55.5% (191/344) among the control group. 79% of the subjects (335/424) had complete data available (a returned questionnaire and a returned MEMs^®^): 44.2% (148/335) among the SMS group, and 55.8% (187/335) among the control group. Figure 1 summarizes completeness of returned MEMs^® ^and questionnaires among both groups by demographic strata.

Demographic characteristics did not differ among those for whom questionnaires were returned and the others (Table 1). The mean age did not differ significantly between groups (26.4 years among the group with completed data, versus 26.7 years, p = 0.47). No significant differences in demographic characteristics were observed among those with returned questionnaires between the SMS and control group (n = 344, 153 in the SMS group and 191 in the control group).

**Table 1:** Demographic characteristics according to completeness of study data (n = 424, France, 2007).

**Complete dataset**		**Incomplete dataset**		**Total**		**P value**	
				
	**n**	**%**	**n**	**%**	**n**	**%**	
Gender							
Male	322	96.1	87	97.8	409	96.5	
Female	13	3.9	2	2.2	15	3.5	
Total	335	100.0	89	100.0	424	100.0	0.74
Rank							
Officer	16	4.8	8	9.0	24	5.7	0.33
NCO and corporal	136	40.6	34	38.2	170	40.1	
Private	183	54.6	47	52.8	230	54.2	
Total	335	100.0	89	100.0	424	100.0	

NCO: Non Commissioned Officers

### Acceptability and feasibility

Acceptability was assessed among the 344 subjects with returned questionnaire. Subjects among the SMS group agreed more frequently that SMS reminders were very useful (52.3% versus 35.1% in the control group, p = 0.001) and that receiving SMS reminders was not annoying (62.8% versus 46.0%, p = 0.02). Additionally, 58.2% among the SMS group agreed that it would be very useful to generalize use of SMS reminders to all soldiers returning from malaria endemic areas, versus 39.9% among the control group (Table 2).

**Table 2:** Subject responses regarding the reminder SMS by study group (n = 344, France, 2007).

	**SMS group**		**Control group**		**Total**		**P value**
				
	**n**	**%**	**n**	**%**	**n**	**%**	
Usefulness of the device							
Very useful	80	52.3	66	35.1	146	42.8	0,01
Mostly useful	51	33.3	91	48.4	142	41.6	
Somewhat useful	14	9.2	17	9.0	31	9.1	
Not useful	8	5.2	14	7.5	22	6.5	
Total	153	100.0	188	100.0	341	100.0	
Laboriousness of the device							
Not annoying	96	62.8	86	46.0	182	53.5	<10^-4^
Somewhat annoying	42	27.4	67	35.8	109	32.1	
Mostly annoying	8	5.2	24	12.8	32	9.4	
Very annoying	7	4.6	10	5.4	17	5.0	
Total	153	100.0	187	100.0	340	100.0	
Usefulness of the generalizing the use of SMS reminders							
Very useful	89	58.2	75	39.9	164	48.1	<10^-4^
Mostly useful	45	29.4	81	43.1	126	37.0	
Somewhat useful	15	9.8	27	14.4	42	12.3	
Not useful	4	2.6	5	2.6	9	2.6	
Total	153	100.0	188	100.0	341	100.0	

Feasibility was evaluated only among the 152 subjects of the SMS group with returned questionnaires. 90.1% (137/152) reported receiving a daily SMS at midday during throughout the study.

### Compliance with malaria chemoprophylaxis

**Figure 2:**
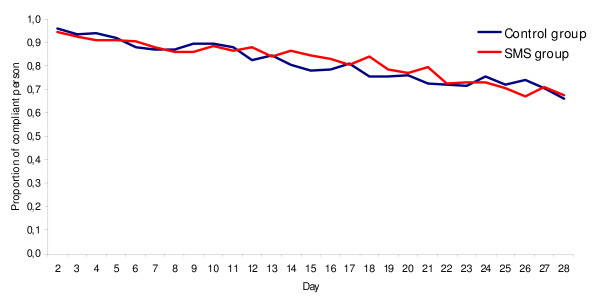
**Daily proportion of compliant persons from day 2 and day 28 in the SMS group and the control group (n = 335, France, 2007)**.

Assessment of compliance with malaria chemoprophylaxis among the 335 subjects with complete data (returned questionnaire and MEMs^®^) revealed the overall daily proportion of compliant subjects decreased from 95.2% (95%CI = 92.4% - 97.2%) to 66.6% (95%CI = 61.2% - 71.6%) between D+2 and D+28. Among subjects assigned to the SMS group, compliance decreased from 94.6% (95%CI = 89.6% - 97.6%) to 67.6% (95%CI = 59.9% - 71.6%). Among subjects assigned to the control group, it decreased from 95.2% (95%CI = 91.8% - 98.1%) to 65.8% (95%CI = 59.0% - 72.5%) (Figure 2). There was no significant difference between groups in the daily proportion of compliant individuals at D+2 and D+28. The others chosen indicators also did not vary significantly between groups (Table 3).

**Table 3:** Compliance indicators in the SMS group and the control group (n = 335, France, 2007).

**Compliance indicators**	**SMS group**		**Control group**		**Total**		**P value**
				
	**n**	**%**	**n**	**%**	**n**	**%**	
One pill per day during the whole study							
Yes	33	22.3	40	21.4	73	21.8	0.84
No	115	77.7	147	78.6	262	78.2	
Total	148	100.0	187	100.0	335	100.0	
Early discontinuation							
Yes	63	42.6	94	50.3	157	46.9	0.16
No	85	57.4	93	49.7	178	53.1	
Total	148	100.0	187	100.0	335	100.0	

**Figure 3:**
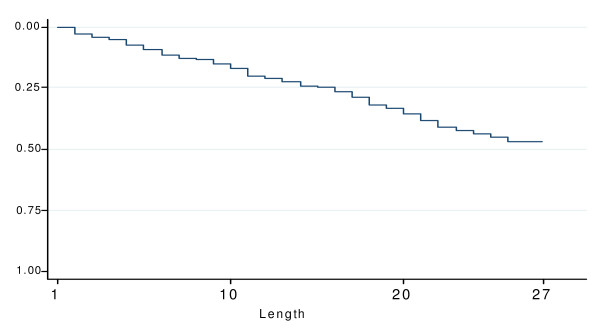
**Probability of early ending between the day 2 and day 28 in the SMS group and the control group (n = 335, France, 2007)**.

Figure 3 represents the Kaplan-Meier curve of the probability of early discontinuation of chemoprophylaxis. In univariate analysis, three variables were associated with early discontinuation: age, previous stay in malaria endemic area and reminder by another person (Table 4). Although assignment to the SMS group was not significantly associated with early discontinuation in univariate analysis; this variable was forced in a multivariate model which demonstrated that subjects who never had been in malaria endemic area were at 1.45 fold higher risk of early ending than the others (RR 95%CI = 1.05 - 2.00). Subjects reminded by someone to take chemoprophylaxis were at 1.43 higher risk of early discontinuation than the others (RR 95%CI = 1.04 - 1.96).

**Table 4:** Determinants of early discontinuation, univariate and multivariate analyse, model of Weibull (n = 335, France, 2007).

	**n subjects**	**% of early ending**	RR*	**95%IC**	**p**	**RR adjusted**	**95%IC**	**P value**
SMS								
Yes	148	42.6	1.00			1.00		
No	187	50.3	1.29	0.94-1.77	0.118	1.22	0.89-1.69	0.218
Age								
≥ 25 years	158	39.9	1.00					
< 25 years	177	53.1	1.50	1.09-2.06	0.013			
Gender								
Male	13	30.8	1.00					
Female	322	47.5	1.68	0.62-4.54	0.305			
Rank								
Officer	16	18.8	1.00					
NCO and corporal	136	46.3	2.86	0.89-9.10				
Private	183	49.7	3.30	1.04-10.43	0.042			
Previous stay in a malaria endemic area								
Yes	216	42.6	1.00			1.00		
No	119	54.6	1.42	1.04-1.96	0.029	1.45	1.05-2.00	0.024
Reminder by another person								
Yes	158	54.4	1.00			1.00		
No	177	40.1	0.66	0.48-0.90	0.010	0.70	0.51-0.96	0.029

### Comparison with malaria chemoprophylaxis compliance assessed in 2004

The total number of taken pills was higher in the control group in the study performed in 2007 than in the cohort studied in 2004 (21.9 and 16.7, respectively, p < 0.001). The proportion of persons who took a daily pill every day was four times lower in the 2004 study than in the 2007 study, although the proportion of early discontinuation did not vary significantly (Table 5). Additionally, the daily proportion of compliance was lower. In 2007, it decreased from 95.2% (95%CI = 91.8% - 98.1%) to 65.8% (95%CI = 59.0% - 72.5%) whereas it decreased from 74.8% (95%CI = 66.4% - 83.1%) to 28.4% (95%CI = 19.7% - 37.2%) in 2004.

**Table 5:** Compliance indicators in the control group in 2007, and the study population in 2004 (France).

	**Control group 2007**		**Whole study population 2004**		**p value**
			
	**n**	**%**	**n**	**%**	
One pill per day during the whole study					
Yes	40	21.4	6	5.2	10^-5^
No	147	78.6	110	94.8	
Total	187	100.0	116	100.0	
Early discontinuation					
Yes	94	50.3	53	45.7	0.48
No	93	49.7	63	54.3	
Total	187	100.0	116	100.0	

## Discussion

### Limitations of the study

Analysis of compliance was limited to approximately 80% of enrolled subjects. The high number of subjects lost to follow-up can be explained by subjects' mobility upon return; some soldiers were transferred to another battalion during the study period, and some others were returned to duty prior to the end of the study period.

Of note, gender and age, characteristics known to be associated to compliance, did not vary significantly between the lost to follow-up and those with returned questionnaire and MEMs. Results should be representative for the 424 soldiers included in the study.

### Acceptability and feasibility

The impact of sending SMS has been previously evaluated in the management of asthma and diabetes [8,9]. SMS reminders have demonstrated efficacy in increasing compliance with vaccination schedules [10] and for improving attendance at health promotion centers or clinics [11,12]. However, the context of malaria chemoprophylaxis taken after returning from a malaria endemic area may be quite different: travelers or soldiers are typically healthy people who usually make a trip for a short period.

This is the first published study aiming to assess the effect of SMS on malaria chemoprophylaxis compliance following return from malaria endemic area. The study demonstrated the feasibility of the SMS reminders for malaria chemoprophylaxis. Some people in the SMS group did not receive a daily SMS reminder because they had given an incorrect mobile device number, or had stayed in an area where mobile service was unavailable.

It is plausible that, after four months spent far away from their relatives and friends, the reminder effect of SMS messages in private life may have been perceived as intrusive and may have contributed to noncompliance. Yet, reassuringly, this study demonstrated that the proportion of people agreeing with the statements that the device was very useful and that generalizing its use to all soldiers returning from duty in malaria endemic area would be very useful, was higher in the group assigned to receive SMS reminders than the control group. Furthermore, the proportion of subjects agreeing that the device was not laborious was higher in the SMS group than in the control group. Subjects who had received SMS reminders overall seemed satisfied. Therefore, the use of SMS reminders could be generalized to all soldiers returning from malaria endemic areas, despite reluctance of those who did not have SMS.

### Impact of SMS on malaria compliance

In 2006, 201 malaria cases occurred among the 11 190 French soldiers deployed to Côte d'Ivoire for a four-month tour. The corresponding malaria incidence rate was 1.8 cases per 100 persons per four months in Côte d'Ivoire. With the same incidence rate, no more than six malaria attacks were expected among the 335 persons with complete data. Considering this very low incidence, a decrease in incidence rate could not be chosen as indicator of compliance. Indeed, only, one malaria attack occurred during the study period among each group. The corresponding calculated incidence (6.7/1,000 persons in the SMS group versus 5.3/1000 persons in the control group) was not statistically significant.

None of the chosen indicators demonstrated improved chemoprophylaxis compliance among subjects assigned to the SMS group. These results can be best explained by the fact that subjects from both the SMS and control group maintained frequent contact with each other during the study period. Unlike in earlier cohorts where many soldiers departed for holidays following their return, among members of this study, many remained at their regiment, where most subjects collectively had lunch at the mess. SMS reminder messages were sent at midday, as a result the reminder by SMS was likely noted not only by subjects enrolled to the SMS group, but potentially also to members enrolled to the control group who had lunch in the same place. This fact may also explain why the overall compliance with malaria chemoprophylaxis was higher than expected. In this study, at D+28, at the end of the study, 66.6% of all subjects remained compliant with malaria chemoprophylaxis. In an earlier study, from 2006, investigation into an outbreak which occurred in another French battalion returning from a malaria endemic area demonstrated that among those who did not have malaria attack, compliance was only 36.6% (95%CI = 27.6%-46.6%) at D+22, based upon measurement of doxycycline plasma concentrations [5]. The validity of comparisons across studies might be affected by differences in ascertainment of compliance (MEMs^® ^versus direct measurement of serum concentrations).

A comparison made with historical data collected in 2004 and that collected in the group without SMS in 2007 demonstrated that the total number of pills taken and the daily proportion of compliance were higher in the control group of the 2007 study than in the earlier study. This suggests those in the 2007 control group may have benefited from incidental exposure to the SMS reminder messages, although this effect cannot be conclusively demonstrated by the methods of this study. Moreover, soldiers themselves considered the reminder by SMS as effective and agreed that its use should be generalized.

Other factors could have interfered with compliance, including the use of MEMs^®^, although these devices were previously used in 2004 following the death of a 22 year old private from severe malaria in 2007 after a stay in Côte d'Ivoire underscored the importance of malaria prevention during and following a stay in a malaria endemic area [6].

### Impact of a person remembering to take the malariachemoprophylaxis

In multivariate analysis, subjects who were reminded by others to take their malaria chemoprophylaxis were at significantly higher risk of early discontinuation. This paradoxical effect may be explained by the fact that persons who never forgot their chemoprophylaxis may not have been perceived by others as needing help, whereas those who forgot were perceived by others as needing help, without this help being sufficient to affect a high level of compliance.

## Conclusion

According to market studies, mobile devices had nearly a 89% penetration rate in France [13] during the period of the study, and it is reasonable to conclude this figure may be higher now. Therefore, with this high penetration rate, sending a daily SMS after returning from a malaria endemic area is feasible, and the results of this study demonstrate is it acceptable. Although this particular study demonstrated that the use of SMS reminder messages did not increase the malaria compliance in comparison to the other group, this is probably because the population under study consisted of soldiers who, in this particular instance stayed together following their return from a malaria endemic area, instead of going on holidays. This weakened the measured effect of the SMS intervention in comparison to the control group. Another study should be done to confirm these results among soldiers going on holidays following return, or with individual travellers.

## Competing interests

The authors declare that they have no competing interests.

## Authors' contributions

LO conceived the study, collected the data, performed the statistical analysis, coordinated the study and wrote the manuscript. OR participated in the design of the study, collected the data, helped in writing the manuscript. CM participated in the design of the study, helped in the statistical analysis and helped in writing the manuscript. RM participated in the design of the study, helped in the statistical analysis, and helped in writing the manuscript. CP collected data and facilitated the study. AT participated in the conception of the study. RM participated in the conception of the study. DB participated in the conception of the study and was the military authority who facilitated the performance of the study. JPB participated in the conception of the study, and its coordination. All authors have read and approved the final manuscript.
